# *In Vitro* Antistaphylococcal Effects of *Embelia schimperi* Extracts and Their Component Embelin with Oxacillin and Tetracycline

**DOI:** 10.1155/2015/175983

**Published:** 2015-02-23

**Authors:** Johana Rondevaldova, Olga Leuner, Alemtshay Teka, Ermias Lulekal, Jaroslav Havlik, Patrick Van Damme, Ladislav Kokoska

**Affiliations:** ^1^Department of Crop Sciences and Agroforestry, Faculty of Tropical AgriSciences, Czech University of Life Sciences Prague, Kamycka 129, Suchdol, 165 21 Prague 6, Czech Republic; ^2^Laboratory for Tropical and Subtropical Agriculture and Ethnobotany, Department of Plant Production, Faculty of Bio-Science Engineering, Ghent University, Coupure links 653, 9000 Ghent, Belgium; ^3^Department of Plant Biology and Biodiversity Management, College of Natural Sciences, Addis Ababa University, P.O. Box 3434, Addis Ababa, Ethiopia; ^4^Department of Biology, College of Natural Sciences, 445 Debre Berhan University, Debre Berhan, Ethiopia; ^5^Department of Microbiology, Nutrition and Dietetics, Faculty of Agrobiology, Food and Natural Resources, Czech University of Life Sciences Prague, Kamycka 129, Suchdol, 165 21 Prague 6, Czech Republic

## Abstract

Bacterial infections are in less-developed countries traditionally treated by remedies prepared from medicinal plants. *Embelia schimperi* (Vatke) is a plant used as a taenicide or disinfectant in Ethiopia, very often taken mixed with another plant species. In the present study, we examined two extracts prepared from seeds and twigs with leaves of *E. schimperi* and its main present secondary metabolite embelin for their antibacterial combinatory effect with oxacillin and tetracycline against sensitive and resistant *Staphylococcus aureus* strains. Minimum inhibitory concentrations were determined through the broth microdilution method, whereas the combinatory effect was evaluated through fractional inhibitory concentration sum (ΣFIC) indices. Results show many positive interactions and synergy occurring in embelin and oxacillin combinations against 4 out of 9 strains (ΣFIC 0.203–0.477) and for embelin and tetracycline combination against 3 out of 9 strains (ΣFIC 0.400–0.496). Moreover, the resistance to oxacillin has been overcome in 2 strains and to tetracycline in 3 strains. According to our knowledge, this is the first study showing antimicrobial combinatory effect of *E. schimperi* as well as of embelin. These findings can be used for the further research targeted on the development of new antistaphylococcal agents.

## 1. Introduction

*Staphylococcus aureus* (sometimes called golden staph) is one of the most serious human pathogens, responsible for dangerous community- and hospital-acquired infections. Most of its strains are resistant to *β*-lactams as well as to other classes of antibiotics, such as tetracyclines [1]. Although mortality and morbidity associated with* S. aureus* infections in less-developed countries far exceed those occurring in developed ones, staphylococcal diseases in low-income countries are still perceived as trivial in comparison with other infections, such as malaria or HIV [2]. Even though common antibiotics can still manage this pathogen, many of them are no longer effective against majority of staphylococcal strains (such as penicillin or tetracycline). Spread of antibiotic resistant strains now has become an important public health problem worldwide [3]. Thus, it is crucial to use new strategies to overcome complications in the treatment of staphylococcal infections caused by drug-resistant strains, such as combinatory antimicrobial effect of plant-derived products and commonly used therapeutics [4].

Medicinal plant extracts play an important role in the traditional treatment of many diseases including bacterial infections. It is well-known that indigenous healers not only use remedies prepared from one single plant species but also prepare specific mixtures of different medicinal plants to treat, for example, oral diseases, wounds, and skin disorders. This traditional medical knowledge is useful not only for community healthcare but also for future drug development [5, 6]. In previous studies, many plant extracts or natural compounds have been shown to possess combinatory antimicrobial activity, including improving antibiotic's efficacy against* S. aureus* [7, 8].

Different plant parts such as fruits, seeds, or roots of* Embelia schimperi* (Vatke), a scandent or climbing shrub widespread in highlands of tropical Africa belonging to the family Myrsinaceae, are traditionally used as an antibacterial and anthelminthic remedy, especially against tapeworm and diarrhea. They are also useful against fevers and chest and skin diseases [6, 9]. A gum obtained from the plant is used as a warming remedy in the treatment of dysmenorrhea. In Ethiopia, the bark or fruits from* E. schimperi* are combined with other species, such an* Albizia anthelmintica*,* Guizotia abyssinica*,* Glinus lotoides*, and* Hagenia abyssinica*, mixed with water and taken as a taenicide or used as a disinfectant [10] which suggests that the pharmacological effect of this plant can be increased by interaction with other plant ingredients.

Although the antibacterial activity of* E. schimperi* has been discussed by several authors [9, 11, 12], there is no report that focuses on its antimicrobial combinatory effect with other herbal or pharmaceutical agents. Therefore, in the present study we evaluated two ethanol extracts prepared from different* E. schimperi* parts and embelin (known also as embelic acid or emberine), the main constituent of the extracts identified by high performance liquid chromatography (HPLC) assay, for their* in vitro* antistaphylococcal combinatory effect with representatives of two typical antibiotic classes associated with staphylococcal resistance.

## 2. Materials and Methods

### 2.1. Plant Material

Plant material of* E. schimperi* was collected and identified by Dr. Lulekal at Ankober District, North Shewa Zone, Amhara Region, Ethiopia. Specimen identification was performed both in the field and at the National Herbarium of Ethiopia (ETH) using taxonomic keys and floras [13, 14] and by comparison with voucher reference herbarium specimens. The collection number of identified voucher specimens deposited at the ETH is ErmiasL505.

### 2.2. Preparation of Extracts

Two kinds of ethanol extracts were prepared, from seeds (extract 1) and from twigs with leaves (extract 2). About 15 g of the air-dried plant material was finely ground using a Grindomix apparatus (GM100 Retsch, Haan, DE) and extracted in 80% ethanol using a laboratory shaker for 24 h. All operations were carried out at room temperature. Each extract was subsequently filtered and concentrated to dryness using a rotary evaporator R-200 (Buchi, CH) in vacuum at 40°C. The extraction yield for seeds was 25.5% and for twigs with leaves 38.5%. Extracts were then dissolved in dimethyl sulfoxide (DMSO) to create a stock solution of 51.2 mg/mL concentration of each extract that was stored at −20°C until tested.

### 2.3. Chemicals

Embelin (2,5-dihydroxy-3-undecyl-1,4-benzoquinone), oxacillin, and tetracycline were obtained from Sigma-Aldrich (Prague, CZ). DMSO (Penta, Prague, CZ), ethanol (Sigma-Aldrich, Prague, CZ), and deionized water were used as solvents for the preparation of stock solutions of antibiotics and embelin (at 100 times higher concentration than the highest concentration tested). Methanol and trifluoroacetic acid (TFA), used as the mobile phase in HPLC assay, were purchased from Lachner (Neratovice, CZ) and Sigma-Aldrich (Prague, CZ), respectively.

### 2.4. HPLC Analysis

HPLC method coupled with an UV-visible diode array detector (DAD) was used for the determination and quantification of embelin in extracts. The separation of compounds in extracts was carried out using a Dionex Summit (Dionex Corp., Sunnyvale, CA, USA) system equipped with a P680 quaternary gradient pump unit, TCC-100 thermostated column compartment, and DAD UV 340U, interfaced with Waters 717 autosampler (Waters Corp., Milford, MA, USA). A Gemini C18 column, 5 *μ*m, 110A, and 4.6 × 250 mm (Phenomenex, Torrance, CA, USA), was used and the column temperature was set to 35°C. Binary gradient elution was performed using 100% methanol and 0.1% TFA as the mobile phase at a ratio of 20 : 80 rising to 100 : 0 over 140 min; flow rate was 0.8 mL min^−1^. UV detection was performed at 260 nm. Data were collected and processed in a Chromeleon data station (version 6.7). The stock solution of standard embelin (1000 *μ*g/mL) was prepared in methanol.

**Figure 1:**
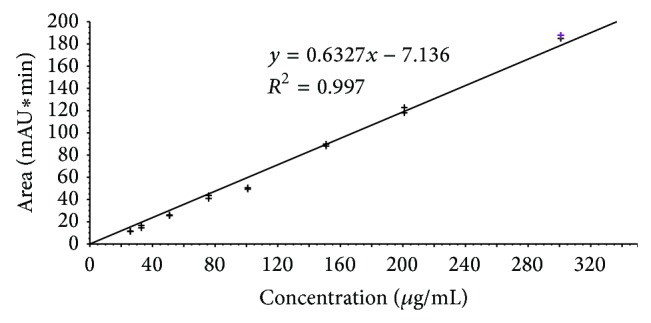
Calibration curve used for quantitative analysis of embelin.

**Figure 2:**
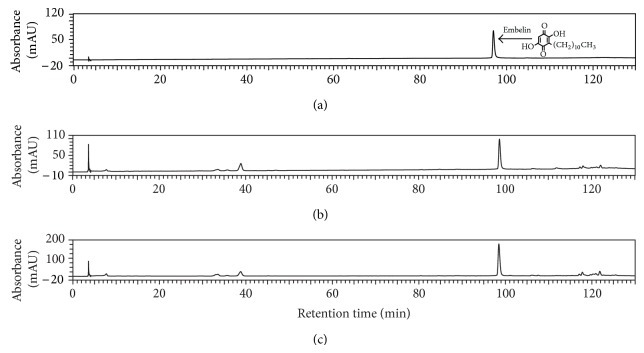
HPLC chromatograms of standard embelin (a) and* Embelia schimperi* ethanol extracts from seeds (b) and from twigs with leaves (c) at 260 nm.

For the determination of embelin, following concentrations were used: 51.2 mg/mL and 25.6 mg/mL of plant extracts and 0.5 mg/mL and 1 mg/mL of standard embelin. Subsequently, series of standard embelin solutions of concentrations 25, 32.5, 50, 75, 100, 150, 200, and 300 *μ*g/mL were analyzed to create an external calibration curve and triplicate injection of 2.56 mg/mL of both extracts was performed to enable the quantification of embelin (Figures 1 and 2). Both determination and quantification were based on triplicate sample preparation. Limit of detection (LOD) and limit of quantification (LOQ) were estimated from the signal-to-noise ratio and were found to be 3.81 and 6.35 *μ*g/mL, respectively.

### 2.5. Bacterial Strains and Growth Media

In this study, antibiotic-sensitive as well as antibiotic-resistant (methicillin-, tetracycline-, and multidrug-resistant) strains were tested. Three standard* S. aureus* strains ATCC 29213, ATCC 43300, and ATCC 33591 disposed at ready-to-use suspension (Culti-Loop) were purchased from Oxoid (Basingstoke, UK). Seven clinical isolates (methicillin-resistant* S. aureus*, MRSA1 and MRSA4; epidemic MRSA, EMRSA15; multidrug-resistant* S. aureus*, MdRSA2 and MdRSA3; and tetracycline-resistant* S. aureus*, TRSA1 and TRSA2) were provided on agar plates from the University Hospital in Motol (Prague, CZ). Bacteria were stored at 4°C until use. Overnight cultures of each strain were directly suspended in 10 mL Mueller–Hinton broth (Oxoid, Basingstoke, UK) equilibrated with Tris-buffered saline (Sigma-Aldrich, Prague, CZ). The turbidity of the bacterial suspension was adjusted to 0.5 McFarland standard (which represents 1.5 × 10^8^ CFU/mL) using Densi-La-Meter II (Lachema, Brno, CZ). As control strain for antibiotic susceptibility testing,* S. aureus *ATCC 29213 was used.

### 2.6. Determination of Minimum Inhibitory Concentrations (MICs) and Evaluation of Combinatory Antimicrobial Effect

MICs were determined by the broth microdilution method described by the Clinical and Laboratory Standards Institute (CLSI) [15], modified according to the recommendations proposed for effective assessment of the anti-infective potential of natural products [16]. Fractional inhibitory concentrations (FIC) were evaluated by the checkerboard assays [17]. The stock solutions of each tested extract or compound were diluted with Mueller-Hinton broth to obtain the starting concentration. The initial concentrations used in combinations were 256 and 4 *μ*g/mL for extracts and embelin, respectively. In the case of antibiotics, the starting concentration was their MIC value. In combinations, twofold serial dilutions of antibiotics prepared in horizontal rows of microtiter plate were subsequently cross-diluted vertically by twofold serial dilutions of the extracts or embelin. After dilution plates were inoculated by the respective bacterial suspension (final density 5 × 10^5^ CFU/mL) and incubated for 24 h at 37°C. Bacterial growth was measured at 405 nm as turbidity by Multiscan Ascent Microplate Photometer (Thermo Fisher Scientific, Waltham, USA). MICs were expressed as the lowest concentrations that inhibited bacterial growth by ≥80% compared with that of the agent-free growth control. The solvents (1%), used as the negative control, did not inhibit any strain tested. All results are presented as the average of MICs obtained from three independent experiments that were performed in triplicate.

The combinatory effects were then determined based on ΣFIC. For combination of compound A and compound B, the ΣFIC is calculated according to the following equation:

(1) $$\begin{array}{c} \Sigma \text{FIC}={\text{FIC}}_{\text{A}}+{\text{FIC}}_{\text{B}}, \end{array}$$

where FIC_A_ = MIC_A(in  the  presence  of  B)_/MIC_A(alone)_ and FIC_B_ = MIC_B(in  the  presence  of  A)_/MIC_B  (alone)_. The results were evaluated according to The European Committee on Antimicrobial Susceptibility Testing [18] as follows: synergistic effect if ΣFIC ≤ 0.5; additive if ΣFIC > 0.5 and ≤ 1; indifferent if ΣFIC > 1 and < 2; and antagonistic if ΣFIC ≥ 2.

## 3. Results and Discussion

In our experiments, both extracts of* E. schimperi* showed significant potentiating activity of oxacillin against* S. aureus*; the best results are summarized in Table 1. Additive interactions occurred for extract 1 and oxacillin combination against 2 (ATCC 43300 and MdRSA3) out of 10 strains (ΣFIC 0.751–0.878) and for extract 2 and oxacillin combination against 6 (ATCC 29213, ATCC 33591, MdRSA2, MRSA4, TRSA1, and TRSA2) out of 10 strains tested (ΣFIC 0.506–0.927). Moreover, in few cases we obtained ΣFIC values lower than 0.6 (0.506–0.563), which can be considered as a strong additive effect. In comparison, extract 2 showed significantly better results than extract 1 when combined with oxacillin. In addition, when the 1/2 MIC of extract 2 was used, the resistance to oxacillin (MIC ≥ 4 *μ*g/mL) [15] was overcome in ATCC 33591 and MdRSA2 with a 170- and 106-fold reduction of oxacillin MIC.

*In vitro* antibacterial activity of* E. schimperi* had previously been examined in our laboratory showing the strongest antimicrobial effect in comparison with other plants used in Ethiopian folk medicine for treatment of infectious diseases [9]. Although this species is traditionally applied as an anti-infective remedy in mixtures with other plants [10], there is no report on its* in vitro* antimicrobial effect in combination with other therapeutical agents. Our findings that growth-inhibitory activity of oxacillin is significantly increased when combined with* E. schimperi* extracts against* S. aureus* may support the traditional medicinal use of this plant in mixtures for the treatment of bacterial infections.

**Figure 3:**
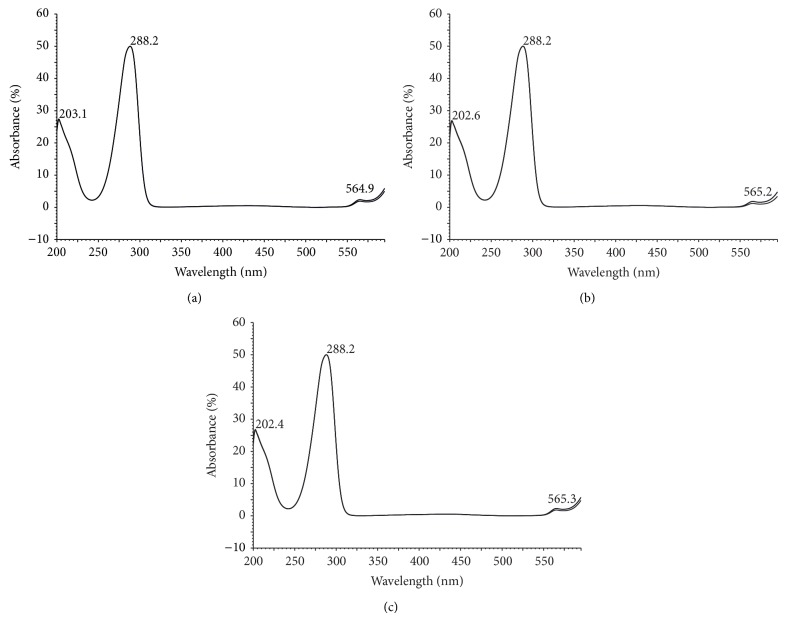
UV spectra of embelin as standard (a) and in extracts from twigs with leaves (b) and seeds (c).

HPLC analysis identified embelin as the main secondary metabolite present in both extracts (Figures 2 and 3). The total content of embelin was 3022.6 *μ*g/mL in extract 2 and 1476.5 *μ*g/mL in extract 1, with a standard deviation of 89.1 and 40.7, corresponding to the content of 1.11% in twigs with leaves and 1.51% in seeds. From the chromatogram profiles, embelin was clearly the most intense peak at 260 nm. These results are consistent with those of Midiwo and Manguro [19] who described presence of embelin in* E. schimperi *and determined its content in fruits, root bark, stem bark, and leaves at relative ratio 1.01–4.31%.

In view of the HPLC analysis, embelin was further investigated for its* in vitro* antistaphylococcal combinatory effect with oxacillin and tetracycline. Individual MICs as well as MICs of its combinations with the corresponding ΣFICs are summarized in Tables 2 and 3. The antimicrobial synergistic effect occurred for the embelin and oxacillin combination against 4 (ATCC 29213, ATCC 43300, EMRSA15, and TRSA2) out of 9 strains tested (ΣFIC 0.203–0.477), and for the embelin and tetracycline combination against 3 (ATCC 43300, MRSA4, and TRSA2) out of 9 strains (ΣFIC 0.400–0.496). The best results (ΣFIC 0.203) were obtained at embelin concentration 2 *μ*g/mL against standard methicillin-resistant* S. aureus *(ATCC 43300). Moreover, our results showed an additive effect of both combinations against most of the strains together with no occurrence of antagonism. Oxacillin resistance (MIC ≥ 4 *μ*g/mL [15]) was overcome in ATCC 43300 at an embelin concentration of 2 *μ*g/mL and in MRSA1 at an embelin concentration of 4 *μ*g/mL causing in both cases a 64-fold reduction of oxacillin MIC. Tetracycline resistance (MIC ≥ 16 *μ*g/mL [15]) was totally broken in all four tetracycline-resistant strains. Moreover, MdRSA2 and MdRSA3 can be considered as multidrug-resistant strains due to their low sensitivity to oxacillin and tetracycline. Our experiments showed that these strains are inhibited more effectively when antibiotics are combined with embelin. Furthermore resistance to tetracycline was overcome in both strains.

Our results on* in vitro* antistaphylococcal effect of embelin with MICs ranging from 8 to 32 *μ*g/mL are consistent with those of a previous study of Radhakrishnan et al. [20] who reported an embelin MIC of 20 *μ*g/mL. In another study, Feresin et al. [21] determined higher MICs against both methicillin-sensitive and methicillin-resistant* S. aureus* strains at 62 and 250 *μ*g/mL, which can be explained by different strains used. However, embelin has been previously described for its synergistic antiproliferation interactions [22–24], but, according to our knowledge, this is the first report of its antimicrobial combinatory activity.

Staphylococcal resistance to *β*-lactams is caused by penicillin-binding proteins with reduced affinity to antibiotics. On the other hand, low sensitivity to tetracycline is connected with a decrease in intracellular accumulation and decreased uptake of drug which is caused by a specific efflux mechanism [25]. There are only a few reports about the mechanism of antibacterial activity of benzoquinones. However, thymoquinone has been shown to cause efflux inhibition and as a result increase intracellular concentration of 4,6-diamidino-2-phenylindole [26]. Therefore we hypothesize that embelin can also act as tetracycline efflux inhibitor in bacterial cells. However, further research focusing on the embelin mode of antistaphylococcal activity when combined with antibiotics is needed for clarification of the mechanism of its synergistic action.

Embelin has been previously reported for a wide spectrum of biological properties, including promotion of wound healing activity and a growth-inhibitory effect against* S. aureus*,* Streptococcus pyogenes*, and* Pseudomonas aeruginosa* [11, 27, 28]. Moreover, there are many* in vivo* studies using animal models which showed no toxic side effects of embelin signifying its safety profile. Poojari et al. [29] observed no significant body weight changes, mortality, or apparent toxic effects on mice that received embelin at doses of 50 mg and 100 mg per kg of body weight/day for 14 days, which is consistent with results of Gupta et al. [30] and Prakash [31]. The results presented here showed that embelin as antistaphylococcal is promising, but further research is required to determine its efficacy in combination with other drugs as antistaphylococcal agent. However, detailed experiments focusing on the toxicological profile of embelin and other antibiotic combinations should be done to determine its safety prior to public use.

## 4. Conclusions

This study showed that* E. schimperi* extracts have marked effect in enhancing the susceptibility of* S. aureus* to oxacillin. Moreover, its main active constituent embelin at subinhibitory concentrations possesses synergy with oxacillin and tetracycline against this bacterium, including its antibiotic-resistant strains. According to our knowledge, this is the first study of antimicrobial combinatory effect of* E. schimperi* as well as of embelin with antibiotics. Generally, these results can be helpful for further research targeting the development of new antistaphylococcal agents especially against resistant forms. However, further research focused on various aspects of the combinatory action of embelin with antibiotics, such as* in vivo* efficacy and toxicological properties, mechanism of action, and delivery techniques will be needed before its possible pharmacological application.

## Figures and Tables

**Table 1:** *In vitro* inhibitory activity of *Embelia schimperi* ethanol extracts in combination with oxacillin against *S. aureus*.

	MICs (*μ*g/mL)		MICs of oxacillin in combination with listed extracts concentrations (*μ*g/mL)									
Extract/strain	Extract	Ox	+256 *μ*g/mL		+128 *μ*g/mL		+64 *μ*g/mL		+32 *μ*g/mL		+16 *μ*g/mL	
			MIC	ΣFIC	MIC	ΣFIC	MIC	ΣFIC	MIC	ΣFIC	MIC	ΣFIC
Seed												
ATCC 43300	341.33	32	0.21	0.757	12.04	0.751	32	1.188	32	1.094	32	1.047
MdRSA3	341.33	64	3	0.797	32.17	0.878	64	1.188	64	1.094	64	1.047
Leaves + twigs												
ATCC 29213	256	0.5	0.01	1.000	0.17	0.844	0.25	0.750	0.25	0.625	0.25	0.563
ATCC 33591	256	341.33	1	1.003	2	0.506	341.33	1.250	341.33	1.125	341.33	1.063
MdRSA2	256	106.67	1	1.009	1	0.509	106.67	1.250	106.67	1.125	106.67	1.063
MRSA4	128	1.5	x	x	0.02	1.010	0.03	0.517	1.42	1.194	1.42	1.069
TRSA1	128	0.5	x	x	0.01	1.020	0.16	0.812	0.25	0.750	0.5	1.125
TRSA2	170.67	1	0.01	1.510	0.02	0.766	0.26	0.630	0.58	0.771	0.83	0.927

MIC: minimum inhibitory concentration expressed as an average from three independent experiments, each performed in triplicate; Ox: oxacillin; ATCC: American type culture collection; MRSA: methicillin-resistant *S. aureus*; TRSA: tetracycline-resistant *S. aureus*; MdRSA: multidrug-resistant *S. aureus*; x: not calculated; ΣFIC: sum of fractional inhibitory concentrations: the combinatory effect is evaluated as follows: synergy if ΣFIC ≤0.5; additive if ΣFIC >0.5 and ≤1; indifferent if ΣFIC >1 and <2; and antagonistic if ΣFIC ≥2.

**Table 2:** *In vitro* inhibitory activity of embelin in combination with oxacillin against *S. aureus*.

	MICs (*μ*g/mL)		MICs of oxacillin in combination with listed embelin concentrations (*μ*g/mL)									
*S. aureus* strain	Emb	Ox	+Emb 4 *μ*g/mL		+Emb 2 *μ*g/mL		+Emb 1 *μ*g/mL		+Emb 0.5 *μ*g/mL		+Emb 0.25 *μ*g/mL	
			MIC	ΣFIC	MIC	ΣFIC	MIC	ΣFIC	MIC	ΣFIC	MIC	ΣFIC
ATCC 29213	32	0.5	0.17	0.458	0.17	0.396	0.17	0.365	0.17	0.349	x	x
ATCC 43300	10.67	21.33	0.33	0.391	0.33	0.203	5.58	0.355	7.33	0.391	13.33	0.648
EMRSA15	10.67	85.33	24.67	0.477	53.33	0.719	74.67	0.922	74.67	0.898	74.67	0.899
MRSA1	8	128	2	0.516	64	0.750	64	0.625	64	0.563	64	0.531
MdRSA2	10.67	341.33	109.33	0.695	256	0.938	341.33	1.094	341.33	1.047	341.33	1.023
MdRSA3	8	341.33	10.67	0.531	266.67	1.031	341.33	1.125	341.33	1.063	341.33	1.031
MRSA4	8	64	64	1.500	64	1.250	64	1.125	64	1.063	64	1.030
TRSA1	8	0.42	0.05	0.631	0.25	0.850	0.33	0.925	0.33	0.863	0.33	0.831
TRSA2	10.67	0.33	0.01	0.398	0.33	1.188	0.33	1.094	0.33	1.047	0.33	1.023

MIC: minimum inhibitory concentration expressed as an average from three independent experiments, each performed in triplicate; Emb: embelin; Ox: oxacillin; ATCC: American type culture collection; MRSA: methicillin-resistant *S. aureus*; EMRSA: epidemic MRSA; TRSA: tetracycline-resistant *S. aureus*; MdRSA: multidrug-resistant *S. aureus*; x: not tested; ΣFIC: sum of fractional inhibitory concentrations: the combinatory effect is evaluated as follows: synergy if ΣFIC ≤0.5; additive if ΣFIC >0.5 and ≤1; indifferent if ΣFIC >1 and <2; and antagonistic if ΣFIC ≥2.

**Table 3:** *In vitro* inhibitory activity of embelin in combination with tetracycline against *S. aureus*.

	MICs (*μ*g/mL)		MICs of tetracycline in combination with listed embelin concentrations (*μ*g/mL)									
*S. aureus* strain	Emb	Tet	+Emb 4 *μ*g/mL		+Emb 2 *μ*g/mL		+Emb 1 *μ*g/mL		+Emb 0.5 *μ*g/mL		+Emb 0.25 *μ*g/mL	
			MIC	ΣFIC	MIC	ΣFIC	MIC	ΣFIC	MIC	ΣFIC	MIC	ΣFIC
ATCC 29213	32	0.33	0.33	1.125	0.33	1.063	0.33	1.031	0.33	1.016	x	x
ATCC 43300	10.67	0.33	0.04	0.496	0.07	0.400	0.17	0.609	0.33	1.047	0.33	1.023
EMRSA15	10.67	0.67	0.14	0.584	0.5	0.934	0.5	0.840	0.58	0.913	0.58	0.889
MRSA1	8	0.25	0.04	0.660	0.13	0.770	0.25	1.125	0.25	1.063	0.25	1.031
MdRSA2	10.67	21.33	10.67	0.875	21.33	1.187	21.33	1.094	21.33	1.047	21.33	1.023
MdRSA3	8	21.33	3	0.641	14	0.906	18.67	1.000	18.67	0.938	18.67	0.907
MRSA4	8	0.25	0.01	0.540	0.04	0.410	0.21	0.965	0.21	0.903	0.21	0.871
TRSA1	8	16	2.33	0.646	9.33	0.833	10.67	0.792	13.33	0.896	13.33	0.864
TRSA2	10.67	16	1.17	0.448	8	0.687	8	0.594	10.67	0.714	10.67	0.690

MIC: minimum inhibitory concentration expressed as an average from three independent experiments, each performed in triplicate; Emb: embelin; Tet: tetracycline; ATCC: American type culture collection; MRSA: methicillin-resistant *S. aureus*; EMRSA: epidemic MRSA; TRSA: tetracycline-resistant *S. aureus*; MdRSA: multidrug-resistant *S. aureus*; x: not tested; ΣFIC: sum of fractional inhibitory concentrations: the combinatory effect is evaluated as follows: synergy if ΣFIC ≤0.5; additive if ΣFIC >0.5 and ≤1; indifferent if ΣFIC >1 and <2; antagonism if ΣFIC ≥2.
